# Association of serum apoA-I with in-stent restenosis in coronary heart disease

**DOI:** 10.1186/s12872-022-02762-y

**Published:** 2022-08-04

**Authors:** Xin Wang, Min Zhang, Jie Cheng, Hua Zhou

**Affiliations:** 1 Department of Cardiology, School of Medicine, East Hospital, Tongji University, Shanghai, China; 2 Department of Cardiology, School of Medicine, Ren Ji Hospital, Shanghai Jiao Tong University, Shanghai, China; 3 Center for Reproductive Medicine, School of Medicine, Ren Ji Hospital, Shanghai Jiao Tong University, Shanghai, China

**Keywords:** In-stent restenosis, apoA-I, Drug-eluting stents, Prediction

## Abstract

**Background:**

Despite use of drug-eluting stents (DES), in-stent restenosis (ISR) continues adversely affecting clinical outcomes of patients undergoing percutaneous coronary intervention (PCI). Apolipoprotein A-I (apoA-I) has athero-protective effects. However, there is a paucity of clinical data regarding the association between apoA-I and ISR. We sought to investigate whether serum apoA-I is related to ISR after DES-based PCI.

**Methods:**

In this retrospective case control study, 604 consecutive patients who underwent DES implantation before were enrolled. Patients who underwent repeat angiography within 12 months were included in the early ISR study (n = 205), while those beyond 12 months were included in the late ISR study (n = 399). ISR was defined as the presence of > 50% diameter stenosis at the stent site or at its edges. Clinical characteristics were compared between ISR and non-ISR patients in the early and late ISR study, respectively, after adjusting for confounding factors by multivariate logistic regression, stratified analysis, and propensity score matching. The predictive value was assessed by univariate and multivariate logistic regression analysis, receiver operating characteristic (ROC) curve analysis, and quartile analysis.

**Results:**

In the early ISR study, 8.8% (18 of 205) patients developed ISR. Serum apoA-I in the ISR group was lower than that in the non-ISR group (1.1 ± 0.26 vs. 1.24 ± 0.23, *P* < 0.05). On multivariate logistic regression analysis, apoA-I was an independent risk factor for early ISR. Incidence of early ISR showed negative correlation with apoA-I and could be predicted by the combined use of apoA-I and glycosylated hemoglobin (HbA1c) level. In the late ISR study, 21.8% (87 of 399) patients developed ISR. On subgroup analysis, late ISR showed negative correlation with apoA-I irrespective of intensive lipid lowering; on multivariate logistic regression analysis, apoA-I was also an independent risk factor for late ISR. In patients with intensive lipid lowering, combined use of apoA-I, stenting time, and diabetes predicted the incidence of late ISR.

**Conclusions:**

ApoA-I was an independent risk factor for ISR, and showed a negative correlation with ISR after DES-based PCI. Combined use of apoA-I and clinical indicators may better predict the incidence of ISR under certain circumstances.

## Background

Despite extensive use of drug-eluting stents (DES), in-stent restenosis (ISR) continues to be a significant problem affecting 5–10% of patients undergoing percutaneous coronary intervention (PCI). ISR has been shown to be an independent risk factor for major adverse cardiac events (MACE) and poor long-term clinical outcomes after DES-based PCI [1–3]. The recent advances in intracoronary imaging, including intravascular ultrasound (IVUS) and optical coherence tomography (OCT), have helped unravel the pathophysiological mechanisms of ISR. Early ISR (i.e., ISR occurring within 12 months after implantation) is mainly caused by vascular endothelial deprivation and neointimal hyperplasia, while late ISR (i.e., ISR occurring beyond 12 months after deployment) is predominantly related to neo-atherosclerosis [4–7]. Previous studies have shown that 3–20% of patients will develop early or late ISR after implantation of new-generation DES together with intensive lipid-lowering therapy [8–12]. This highlights the clinical relevance of preventing ISR.

Apolipoprotein A-I (apoA-I), the major protein component of high-density lipoprotein cholesterol (HDL-C), is mainly involved in cholesterol reverse transport, and plays a pivotal role in the regulation of lipid metabolism. In recent animal studies, apoA-I was shown to decrease the occurrence and progression of neo-atherosclerosis and ISR by inhibiting inflammation, smooth muscle cell (SMC) proliferation, angiogenesis, and platelet activation [13–19]. However, there is a paucity of clinical data regarding the association of apoA-I with ISR in patients with coronary heart disease undergoing PCI with DES implantation. In this study, we sought to investigate whether serum apoA-I is related to early and late ISR after DES-based PCI.

## Methods

### Study population

**Fig. 1:**
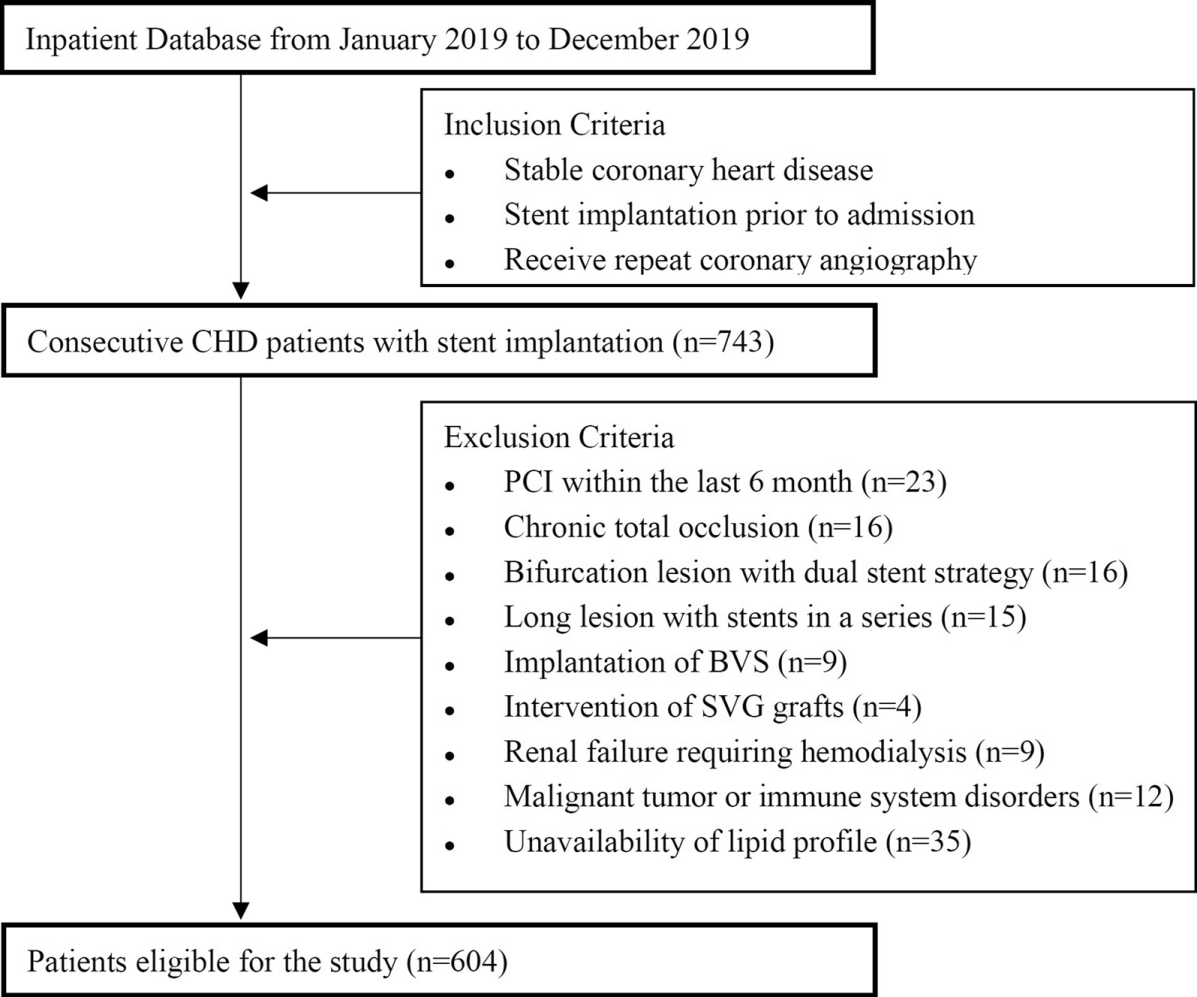
Flowchart of patient enrollment

A total of 743 consecutive patients who received repeat coronary angiography within or beyond 12 months of initial DES-based PCI for de novo coronary lesions, were screened from the database of Shanghai East Hospital from January 2019 to December 2019. Of these, 139 patients were excluded because of the following exclusion criteria: PCI within the last 6 month (n = 23); chronic total occlusion (CTO) (n = 16); bifurcation lesion with dual stent strategy (n = 16); long lesion with stents in a series (n = 15); implantation of biodegradable vascular scaffold (BVS) (n = 9); intervention for saphenous vein graft (SVG) stenosis (n = 4); renal failure requiring hemodialysis (n = 9); malignant tumor or immune system disorders (n = 12); and non-availability of lipid profile data (n = 35). Thus, the remaining 604 patients were included in the final analysis. Among these, 205 patients underwent repeat angiography within 12 months of initial DES-based PCI (early ISR study) and 399 underwent repeat angiography beyond 12 months after initial procedure (late ISR study) (Fig. 1).

### Data collection

Demographic and clinical characteristics including age, sex, risk factors for coronary artery disease (smoking, hypertension, diabetes, hyperlipidemia), and medication history were collected. Serum lipid profile and levels of creatinine, high-sensitivity C-reactive protein, and glycosylated hemoglobin (HbA1c) were determined using standard laboratory techniques. Glomerular filtration rate (GFR) was estimated using the Chronic Kidney Disease Epidemiology Collaboration (CKD-EPI) equation [20]. Left ventricular ejection fraction (LVEF) was assessed by two-dimensional echocardiography according to the modified Simpson’s rule.

### Coronary angiography and intervention

The initial coronary angiography and PCI with stent implantation as well as the repeat coronary angiography were performed using the standard technique described previously [21]. Selective coronary angiography was performed in different views (at least two orthogonal views for each segment of the coronary). The choice of DES was at the discretion of the operator. Pre-dilation and post-dilation were routinely performed during stent implantation with standard manipulation. However, nitroglycerin was routinely used to exclude coronary spasm. Initial PCI-related information was summarized, such as stenting time (time from initial PCI), lesion distribution, number of stents used, stent diameter, and stent length. The results of repeat coronary angiography were reviewed by two experienced interventional cardiologists, who were blinded to the patients' clinical data, using quantitative coronary angiography (QCA). ISR was defined as the presence of > 50% diameter stenosis on angiography at the stent site or at its edges (adjacent 5 mm segments).

### Statistical analysis

Normally distributed continuous variables are presented as mean ± standard deviation and between-group differences assessed using two independent samples *t* test. Non-normally distributed continuous variables are presented as median (interquartile range) and between-group differences assessed using the Mann Whitney U-test. Categorical variables are presented as frequency (percentage) and between-group differences assessed using the Chi squared test. Pearson’s correlation was used to assess the correlation between normally distributed continuous variables. Spearman’s correlation was used to assess correlation of stenting time with age, lipid profile, or HbA1c level; Kendall's Tau-b method was employed to assess the correlation between sex, hypertension, diabetes, smoking status, and other categorical variables or between categorical variables and other continuous variables.

Univariate logistic regression was used to identify the risk factors for ISR. Multivariate logistic regression was performed to identify the independent risk factors and odds ratio with 95% confidence interval (CI) were calculated after adjusting for age, sex, hypertension, diabetes, smoking status, and significant variables in the univariate analysis. Receiver operating characteristic (ROC) curve analysis was performed to assess the ability of apoA-I alone or in combination with other significant variables to distinguish between ISR group and non-ISR group; the optimal cut-off values as well as the corresponding area under the curve (AUC) were calculated. Net reclassification index (NRI) and integrated discrimination improvement (IDI) were used to determine the optimal prediction model. P for trend was calculated using quartile analysis to assess any linear correlation between apoA-I and ISR.

Propensity score matching (PSM) was performed in a 1:1 ratio, using different clinical characteristics (such as sex, age, stenting time, hypertension, diabetes, and smoking) and laboratory parameters as matching variables, and using 0.1 or 0.05 as the calipers, according to the sample size. Thus, a new 1:1 case–control study was performed for further analysis.

All statistical analyses were performed using SPSS 22.0 and R Project 2.15.3. Two-tailed *P* values < 0.05 were considered indicative of statistical significance.

## Results

### Association of apoA-I with early ISR

Among the 205 patients who underwent repeat angiography within 12 months after initial PCI, 18 patients developed ISR (ISR group) while 187 patients did not develop ISR (non-ISR group). There were no significant differences between ISR and non-ISR groups with respect to age and sex distribution, proportion of patients with hypertension, smoking, and history of old myocardial infarction; however, ISR group had a higher proportion of patients with diabetes mellitus (78% vs. 32%, *P* < 0.05). There were no significant between-group differences with respect to lipid profile (including LDL-C), serum hs-CRP, creatine, eGFR, LVEF, and medication history. Lesion distribution, number, diameter, and length of stents were also comparable in the two groups. In the ISR group, serum level of apoA-I was significantly lower (1.1 ± 0.26 vs. 1.24 ± 0.23, *P* < 0.05) while HbA1c level was significantly higher (7.65 ± 1.21 vs. 6.81 ± 1.51, *P* < 0.05) than that in the non-ISR group (Table 1).

**Table 1:** Patient characteristics and clinical profiles

Variants	Early-ISR study (n = 205)			Late-ISR study (n = 399)								
	ISR (n = 18)	Non-ISR (n = 187)	*P* value	Overall			LDL-C ≤ 1.8 mmol/L			LDL-C > 1.8 mmol/L		
				ISR (n = 87)	Non-ISR (n = 312)	*P* value	ISR (n = 29)	Non-ISR (n = 156)	*P* value	ISR (n = 58)	Non-ISR (n = 156)	*P* value
Male	13 (72%)	131 (70%)	0.848	68 (78%)	221 (71%)	0.176	25 (86%)	113 (72%)	0.118	43 (74%)	108 (69%)	0.484
Age	67.67 ± 11.6	65.65 ± 9.49	0.399	66.78 ± 9.58	66.78 ± 10.5	0.988	66.97 ± 7.09	66.35 ± 10.2	0.755	66.69 ± 10.67	67.21 ± 10.8	0.752
Hypertension	13 (72%)	114 (61%)	0.361	60 (69%)	232 (74%)	0.394	20 (69%)	117 (75%)	0.690	40 (69%)	115 (74%)	0.490
Diabetes	14 (78%)	60 (32%)	< 0.001*	37 (43%)	108 (35%)	0.151	15 (52%)	56 (36%)	0.077	22 (38%)	52 (33%)	0.597
Old MI	3 (16.7%)	38 (20.3%)	0.711	24 (27%)	61 (20%)	0.043*	7 (24%)	30 (19%)	0.089	17 (29%)	31 (20%)	0.141
Smoking	5 (28%)	47 (25%)	0.816	22 (25%)	62 (20%)	0.251	8 (28%)	32 (21%)	0.341	14 (9%)	30 (19%)	0.431
Total cholesterol	3.34 ± 0.79	3.28 ± 0.96	0.805	3.82 ± 1.39	3.38 ± 0.91	< 0.001*	2.71 ± 0.37	2.81 ± 0.41	0.216	4.37 ± 1.38	3.96 ± 0.91	0.023*
Triglyceride	1.40 ± 1.13	1.54 ± 1.02	0.586	1.83 ± 1.1	1.51 ± 1.14	0.017*	1.52 ± 1.12	1.3 ± 0.97	0.277	1.99 ± 1.06	1.71 ± 1.25	0.149
LDL-C	2.04 ± 0.69	1.92 ± 0.8	0.530	2.45 ± 1.35	2.01 ± 0.81	< 0.001*	1.39 ± 0.23	1.45 ± 0.27	0.253	2.99 ± 1.36	2.56 ± 0.79	0.014*
HDL-C	1.09 ± 0.35	1.12 ± 0.37	0.729	1.02 ± 0.24	1.16 ± 0.32	< 0.001*	1.04 ± 0.26	1.16 ± 0.29	0.050*	1.01 ± 0.23	1.16 ± 0.34	0.002*
apoA-I	1.10 ± 0.26	1.24 ± 0.23	0.039*	1.16 ± 0.19	1.26 ± 0.23	0.002*	1.11 ± 0.18	1.24 ± 0.22	0.011*	1.19 ± 0.2	1.28 ± 0.25	0.022*
apoB	0.74 ± 0.21	0.7 ± 0.23	0.545	0.88 ± 0.39	0.72 ± 0.24	< 0.001*	0.56 ± 0.11	0.55 ± 0.1	0.791	1.03 ± 0.38	0.88 ± 0.24	0.004*
apoE	30.17 ± 16.33	33.44 ± 14.89	0.450	40.31 ± 16.77	35.33 ± 14.84	0.015*	32.85 ± 17.32	31.52 ± 13.73	0.681	43.81 ± 15.47	39.25 ± 14.98	0.082
Lp(a)	23.8 ± 43.05	43.14 ± 56.85	0.233	45.94 ± 65.49	53.83 ± 70.86	0.396	51.53 ± 81.12	48.13 ± 70.09	0.834	43.31 ± 57.49	59.7 ± 71.44	0.156
HbA1c	7.65 ± 1.21	6.81 ± 1.51	0.039*	6.94 ± 1.65	6.55 ± 1.28	0.021*	6.36 ± 0.83	6.44 ± 1.25	0.748	7.23 ± 1.87	6.65 ± 1.3	0.019*
hs-CRP	3.07 ± 3.84	3.04 ± 5.8	0.376	4.04 ± 8.16	4.84 ± 9.17	0.453	3.39 ± 6.43	4.58 ± 8.2	0.414	4.7 ± 9.59	4.97 ± 9.67	0.864
Creatine	78.44 ± 16.65	74.98 ± 17.07	0.432	83.65 ± 29.34	80.52 ± 34.43	0.445	91.52 ± 32.73	81.74 ± 42.66	0.243	79.57 ± 26.82	79.3 ± 23.58	0.943
eGFR	83.92 ± 14.93	88.87 ± 19.61	0.751	83.65 ± 23.68	84.4 ± 21.52	0.781	79.24 ± 22.61	84.46 ± 22.29	0.250	85.94 ± 24.09	84.35 ± 20.79	0.638
LVEF	56.78 ± 11.1	60.21 ± 6.58	0.139	56.98 ± 8.67	59.11 ± 7.42	0.024*	59.33 ± 7.15	57.71 ± 8.91	0.292	56.62 ± 8.61	58.89 ± 7.71	0.065
Stenting time	/			44 (27–96)	32 (19–52)	< 0.001*	35 (24–84)	26 (17–48)	0.004*	48 (28–96)	36 (23–60)	0.002*
Stent number	1.6 ± 0.7	1.6 ± 0.88	0.987	1.64 ± 0.83	1.82 ± 1.15	0.444	1.6 ± 0.7	1.73 ± 1.02	0.691	1.67 ± 0.91	1.87 ± 1.14	0.487
Stent diameter	2.69 ± 0.46	2.89 ± 0.42	0.131	2.78 ± 0.41	2.83 ± 0.44	0.433	2.88 ± 0.38	2.79 ± 0.42	0.432	2.73 ± 0.43	2.89 ± 0.46	0.074
Stent length	25.86 ± 7.68	27.31 ± 7.24	0.716	28.5 ± 9.34	25.05 ± 7.52	0.143	28.5 ± 9.34	24.98 ± 7.52	0.135	27.72 ± 6.08	26.73 ± 8.03	0.536
Target vessel												
LAD	12	130	0.966	58	225	0.669	19	117	0.862	39	108	0.570
LCX	3	44	0.564	20	96	0.440	5	46	0.375	15	50	0.673
RCA	8	73	0.601	32	125	0.824	11	59	0.549	21	66	0.809
Medicine												
Antiplatelet	16	163	0.834	64	229	0.975	22	133	0.208	42	96	0.139
Anticoagulation	2	21	0.988	5	22	0.668	2	14	0.715	3	8	0.990
ACEI/ARB	5	77	0.268	45	173	0.537	13	82	0.444	32	91	0.678
Statin	16	171	0.715	62	257	0.022*	26	147	0.358	36	110	0.238

*MI* myocardial infarction, *LAD* left anterior descending coronary artery, *LCX* left circumflex coronary artery, *RCA* right coronary artery, *ACEI* angiotensin converting enzyme inhibitor, *ARB* angiotensin receptor blocker

On univariate logistic regression analysis, apoA-I, diabetes, and HbA1c level were risk factors for early ISR (Table 2). However, apoA-I level showed no significant correlation with diabetes or HbA1c level by Kendall’s tau-b correlation.

**Table 2:** Results of univariate logistic regression analysis

Variants	Early ISR study			Late ISR study Intensive lipid lowering subgroup		
	*P* value	OR	95% CI	*P* value	OR	95% CI
Male	0.848	0.9	(0.306–2.644)	0.127	0.420	(0.138–1.279)
Age	0.397	1.022	(0.972–1.075)	0.753	1.007	(0.966–1.048)
Hypertension	0.365	1.642	(0.562–4.801)	0.690	0.833	(0.340–2.042)
Diabetes	0.001	7.35	(2.320–23.281)	0.100	1.926	(0.881–4.208)
Old MI	0.712	1.275	(0.351–4.631)	0.485	0.714	(0.278–1.835)
Smoking	0.816	1.137	(0.385–3.360)	0.344	1.550	(0.626–3.841)
Total cholesterol	0.804	1.065	(0.648–1.751)	0.212	0.505	(0.173–1.476)
Triglyceride	0.584	0.851	(0.478–1.516)	0.286	1.201	(0.858–1.683)
LDL-C	0.529	1.202	(0.678–2.131)	0.253	0.434	(0.103–1.819)
HDL-C	0.728	0.774	(0.184–3.261)	0.051	0.197	(0.038–1.009)
apoA-I	0.038*	0.052*	(0.003–0.849)	0.014*	0.046*	(0.004–0.532)
apoB	0.543	2.023	(0.208–19.637)	0.789	1.849	(0.020–166.82)
apoE	0.448	0.982	(0.936–1.030)	0.680	1.006	(0.977–1.036)
Lp(a)	0.243	0.990	(0.973–1.007)	0.833	1.001	(0.995–1.007)
HbA1c	0.049*	1.307*	(1.001–1.706)	0.746	0.945	(0.670–1.333)
hs-CRP	0.984	1.001	(0.917–1.092)	0.309	1.027	(0.976–1.080)
Creatine	0.320	1.013	(0.988–1.039)	0.287	1.004	(0.997–1.012)
eGFR	0.442	0.992	(0.970–1.013)	0.250	0.990	(0.973–1.007)
LVEF	0.171	0.959	(0.903–1.018)	0.292	0.973	(0.926–1.024)
Stenting time	/			0.004*	1.016*	(1.005–1.027)

On multivariate logistic regression analysis, apoA-I (OR 0.013, 95% CI 0.000–0.574) and HbA1c (OR 1.429, 95% CI 1.022–1.999) were independent risk factors for early ISR.

**Fig. 2:**
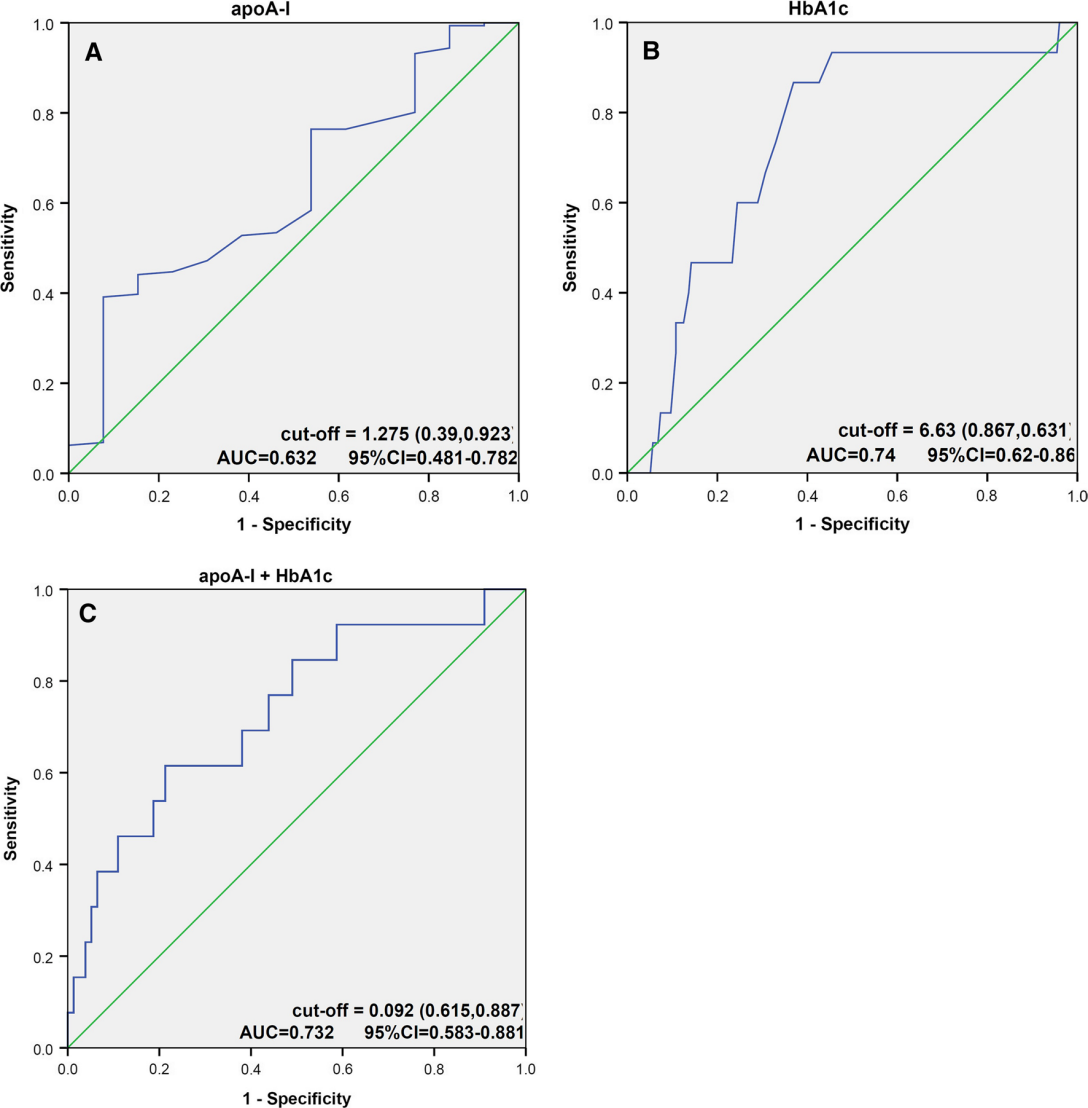
Receiver operating characteristic (ROC) analysis using **A** apoA-I, **B** HbA1c, **C** apoA-I and HbA1c combined, to estimate the strength of the model predicting the incidence of early in-stent restenosis

On ROC curve analysis, the optimal cut-off value of apoA-I (1.275 g/L) had a 39% sensitivity and 92.3% specificity for diagnosis of early ISR (AUC 0.632); however, the AUC for HbA1c alone or combined model (apoA-I, HbA1c) was comparatively higher (Fig. 2). The combined model was associated with a significant absolute NRI of 11.31% (*P* < 0.001) and an IDI of 6.86% (*z* = 1.75, *P* = 0.04) compared with HbA1c alone, suggesting that combined use of apoA-I and HbA1c can better predict the incidence of early ISR.

**Fig. 3:**
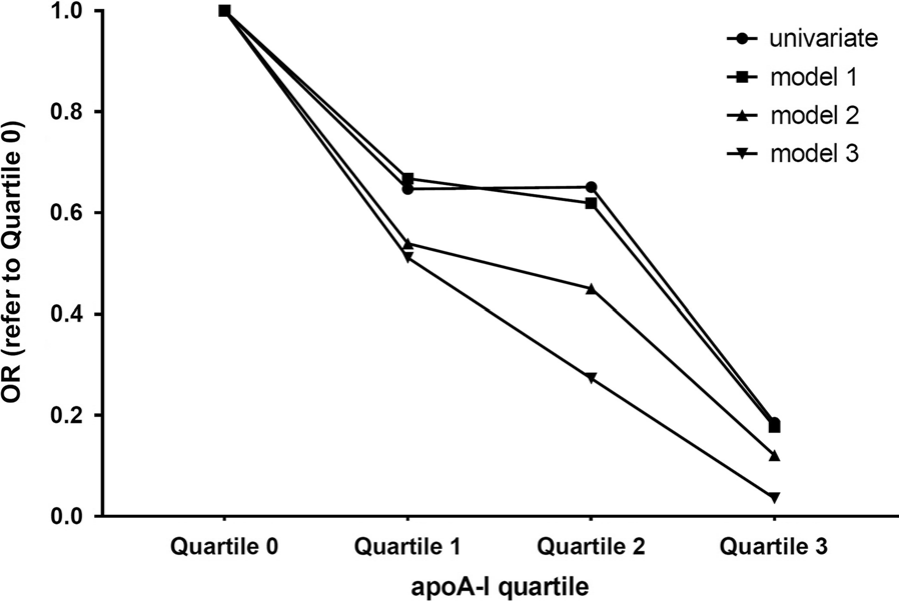
Association of each apoA-I quartile with early ISR incidence, adjusted by three models

With increasing quartile of apoA-I, the incidence of early ISR decreased by 35.3%, 34.9%, and 81.5%, respectively, compared with the lowest quartile. The downward trend was more obvious after adjusting for various confounding factors in the three clinical models (48.9%, 72.7%, 96.4%). These results indicated a negative correlation between the incidence of early ISR and apoA-I (Table 3, Fig. 3).

**Table 3:** Quartile analysis of apoA-I associated with the incidence of early ISR

apoA-I	0–25%	25–50%	50–75%	75–100%	*P* for trend
Quartile	0	1	2	3	
Mean	0.9 ± 0.12	1.13 ± 0.04	1.24 ± 0.03	1.45 ± 0.04	
Num	43	51	38	42	
Non-ISR	38	47	35	41	
ISR	5	4	3	1	
OR	1.00 (ref)	0.647	0.651	0.185	0.509
Model 1	1.00 (ref)	0.668	0.619	0.177	0.560
Model 2	1.00 (ref)	0.539	0.450	0.121	0.595
Model 3	1.00 (ref)	0.511	0.273	0.036	0.324

Model 1: adjusted for age, sex

Model 2: adjusted for age, sex, TC, TG, LDL-C

Model 3: adjusted for age, sex, TC, TG, LDL-C, hypertension, diabetes, smoking

### Association of apoA-I with late ISR

Among the 399 patients who underwent repeat angiography beyond 12 months after initial PCI, 87 patients developed ISR (median stenting time, 44 months) and 312 did not develop ISR (median stenting time, 32 months). There were no significant differences between ISR and non-ISR groups with respect to age, sex, risk factors for coronary artery disease, hs-CRP, creatine, eGFR, coronary lesion distribution, and number/diameter/length of stent (*P* > 0.05 for all). However, the ISR group had a significantly higher prevalence of old myocardial infarction (OMI) (27% vs. 20%), lower LVEF (56.98 ± 8.67 vs. 59.11 ± 7.42), and lower compliance with statin therapy (71.3% vs. 82.4%). Patients with ISR had higher HbA1c level than those with non-ISR (6.94 ± 1.65% vs. 6.55 ± 1.28%, *P* = 0.021). There were significant differences in lipid profile between the two groups; ISR patients had significantly higher levels of total cholesterol (TC) (3.82 ± 1.39 vs. 3.38 ± 0.91), triglycerides (TG) (1.83 ± 1.1 vs. 1.51 ± 1.14), LDL-C (2.45 ± 1.35 vs. 2.01 ± 0.81), apoB (0.88 ± 0.39 vs. 0.72 ± 0.24), apoE (40.31 ± 16.77 vs. 35.33 ± 14.84), but lower levels of HDL-C (1.02 ± 0.24 vs. 1.16 ± 0.32) and apoA-I (1.16 ± 0.19 vs. 1.26 ± 0.23). LDL-C showed a correlation with TC, TG, apoB, and apoE levels, but not with HDL-C or apoA-I levels. Therefore, patients were stratified by LDL-C level for further analysis whereby patients with LDL-C ≤ 1.8 mmol/L were assigned to the intensive lipid-lowering subgroup, while the others (LDL-C > 1.8 mmol/L) were assigned to the non-intensive lipid-lowering subgroup.

### Intensive lipid-lowering subgroup

In the intensive lipid-lowering subgroup, there were no significantly different features between the ISR and non-ISR patients except for median stenting time (35 vs. 26 months), HDL-C (1.04 ± 0.26 vs. 1.16 ± 0.29), and apoA-I (1.11 ± 0.18 vs. 1.24 ± 0.22) (Table 1).

On univariate logistic regression analysis, apoA-I and stenting time were risk factors of late ISR in the intensive lipid-lowering subgroup (Table 2). There was no significant correlation between apoA-I and stenting time.

On multivariate logistic regression analysis, apoA-I (OR 0.037, 95% CI 0.002–0.695), stenting time (OR 1.017, 95% CI 1.003–1.031), and diabetes (OR 2.853, 95% CI 1.027–7.930) were independent risk factors for late ISR after adjusting for sex, age, hypertension, smoking status, TG, LDL-C, hs-CRP, eGFR, LVEF, and other confounding factors.

**Fig. 4:**
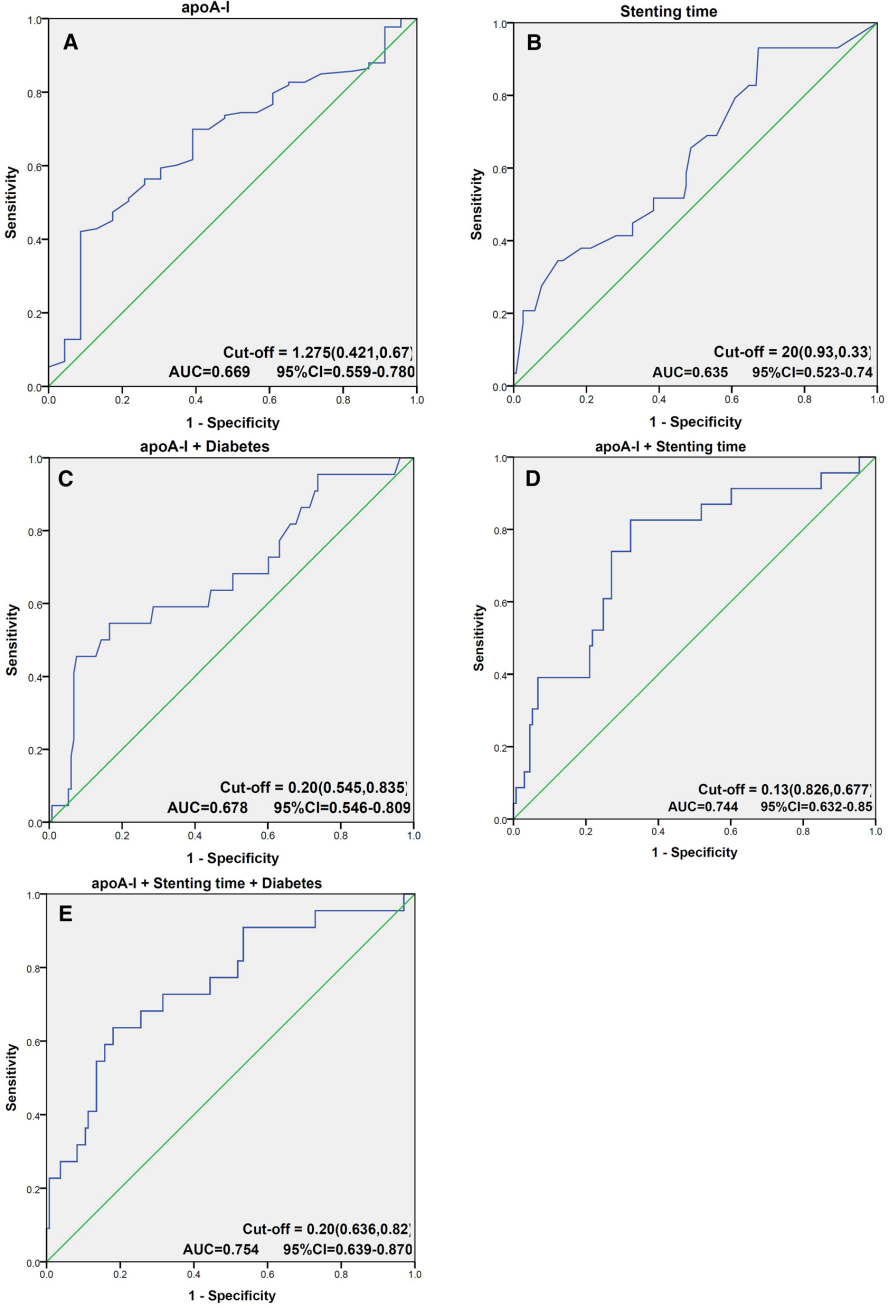
Receiver operating characteristic (ROC) curve analysis using **A** apoA-I, **B** stenting time, **C** apoA-I and diabetes combined, **D** apoA-I and stenting time combined, **E** apoA-I and stenting time and diabetes combined, to estimate the strength of the model predicting the incidence of late in-stent restenosis

ROC curve analysis revealed five clinical models to predict the incidence of late ISR, among which the dual model (apoA-I, stenting time) and triple model (apoA-I, stenting time, diabetes) had comparable higher AUC (0.744 and 0.754) (Fig. 4). Compared with the dual model, the triple model had an additive NRI of − 3.26% (*P* = 0.752), an absolute NRI of 11.04% (*P* = 0.367), but a significant IDI of 4% (*z* = 2.27, *P* = 0.011). This suggested that use of apoA-I together with stenting time and diabetes can better predict the incidence of late ISR.

**Fig. 5:**
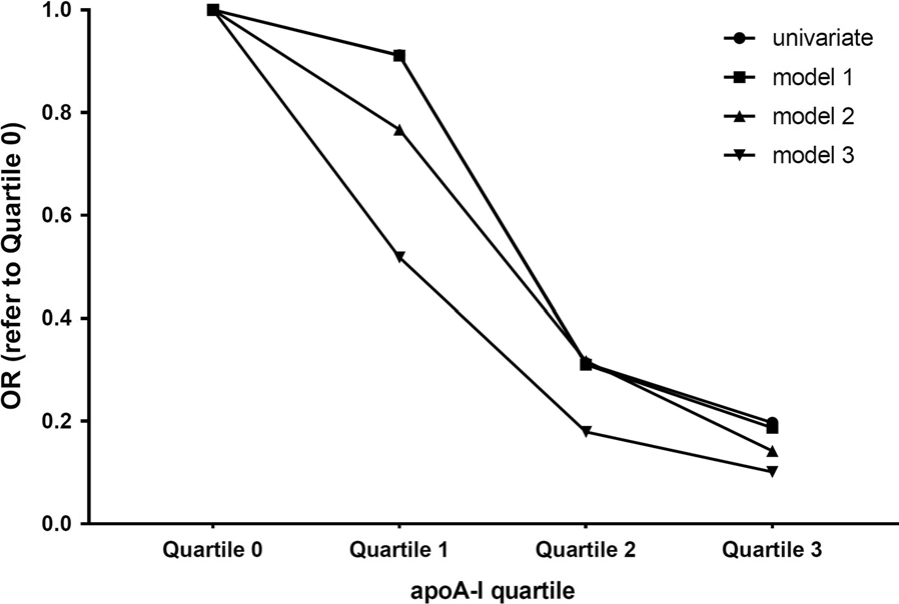
Association of each apoA-I quartile with late ISR incidence in patients with intensive lipid-lowering, adjusted by three models

Quartile analysis showed that with increasing quartile of apoA-I, the incidence of late ISR decreased by 8.8%, 68.7%, and 80.3%, respectively, compared with the lowest quartile. When further adjusted for different confounding factors by three clinical models, the downward trend was more obvious (48.2%, 82.1%, 89.9%). These results showed that the incidence of late ISR in intensive lipid-lowering patients was negatively related with apoA-I (Table 4, Fig. 5).

**Table 4:** Quartile analysis of apoA-I associated with the incidence of late ISR

apoA-I	0–25%	25–50%	50–75%	75–100%	*P* for trend
Quartile	0	1	2	3	
Mean	0.96 ± 0.07	1.13 ± 0.04	1.3 ± 0.06	1.5 ± 0.1	
Num	40	43	36	37	
Non-ISR	31	34	33	35	
ISR	9	9	3	2	
OR	1.00 (ref)	0.912	0.313	0.197	0.102
Model 1	1.00 (ref)	0.911	0.310	0.187	0.127
Model 2	1.00 (ref)	0.767	0.317	0.142	0.117
Model 3	1.00 (ref)	0.518	0.179	0.101	0.318

Model 1: adjusted for age, sex

Model 2: adjustedfor age, sex, TC, TG, LDL-C

Model 3: adjusted for age, sex, TC, TG, LDL-C, hypertension, diabetes, smoking

### Non-intensive lipid-lowering subgroup

In patients without intensive lipid-lowering, differences in lipid profile (including TC, LDL-C, HDL-C, apoA-I, apoB), HbA1c, and stenting time remained statistically significant (Table 1). Therefore, propensity score matching (PSM) was performed in a 1:1 ratio, using sex, age, stenting time, TC, LDL-C, HDL-C, HbA1c, hypertension, diabetes, and smoking as matching variables, and 0.1 as the calipers. As a result, a new case group (ISR-PSM, n = 47) and a control group (NISR-PSM, n = 41) were obtained to evaluate the association of apoA-I with late ISR.

After PSM, ISR patients had significantly lower apoA-I (1.19 ± 0.20 vs. 1.42 ± 0.30) and HDL-C (1.03 ± 0.25 vs. 1.31 ± 0.45) level, while there were no significant differences with respect to the other clinical or laboratory features (Table 5).

**Table 5:** Patient characteristics and clinical profiles after propensity score matching

Variants	ISR-PSM	Non-ISR-PSM	*P* value
	N = 47	N = 41	
Male	34 (72%)	24 (59%)	0.175
Age	65.79 ± 9.55	68.02 ± 12.16	0.423
Hypertension	31 (66%)	26 (63%)	0.803
Diabetes	18 (38%)	13 (32%)	0.519
Old MI	13 (28%)	9 (22%)	0.537
Smoking	12 (25%)	5 (12%)	0.121
Total cholesterol	4.43 ± 1.49	4.65 ± 1.18	0.451
Triglyceride	1.94 ± 1.16	2.07 ± 1.51	0.647
LDL-C	3.06 ± 1.46	3.04 ± 1.09	0.936
HDL-C	1.03 ± 0.25	1.31 ± 0.45	0.002*
apoA-I	1.19 ± 0.2	1.42 ± 0.30	< 0.001*
apoB	1.02 ± 0.38	1.03 ± 0.28	0.980
apoE	43.93 ± 15.79	46.69 ± 19.42	0.462
Lp(a)	39.49 ± 49.19	51.8 ± 64.14	0.312
HbA1c	7.32 ± 1.97	7.07 ± 1.77	0.529
Hs-CRP	5.31 ± 10.62	3.95 ± 6.64	0.519
Creatine	76.54 ± 24.4	77.37 ± 23.57	0.874
eGFR	87.79 ± 22.34	82.3 ± 20.06	0.234
LVEF	59.96 ± 11.09	63.37 ± 9.62	0.132
Stenting time	48 (28–108)	48 (31–84)	0.596
Stent number	1.6 ± 0.74	1.64 ± 0.74	0.877
Stent diameter	2.8 ± 0.44	3.0 ± 0.4	0.121
Stent length	27.46 ± 6.61	28.17 ± 6.37	0.707

On multivariate logistic regression analysis of PSM cohort, apoA-I (OR 0.011, 95% CI 0.000–0.946) was an independent risk factor for late ISR, rather than HDL-C.

**Fig. 6:**
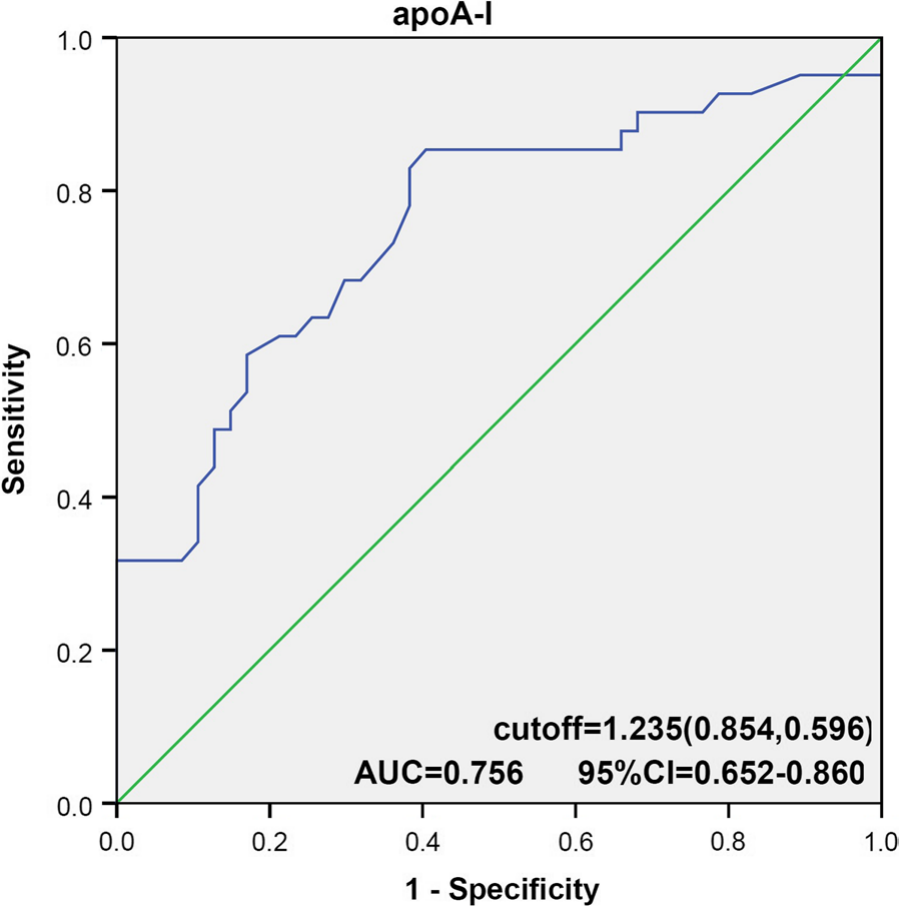
Receiver operating characteristic (ROC) analysis using apoA-I, to estimate the strength of the model predicting the incidence of late in-stent restenosis after PS matching

On ROC curve analysis, the optimal cut-off apoA-I level of 1.235 g/L as the diagnostic criteria was associated with 85.4% sensitivity and 59.6% specificity (AUC 0.756) (Fig. 6).

**Fig. 7:**
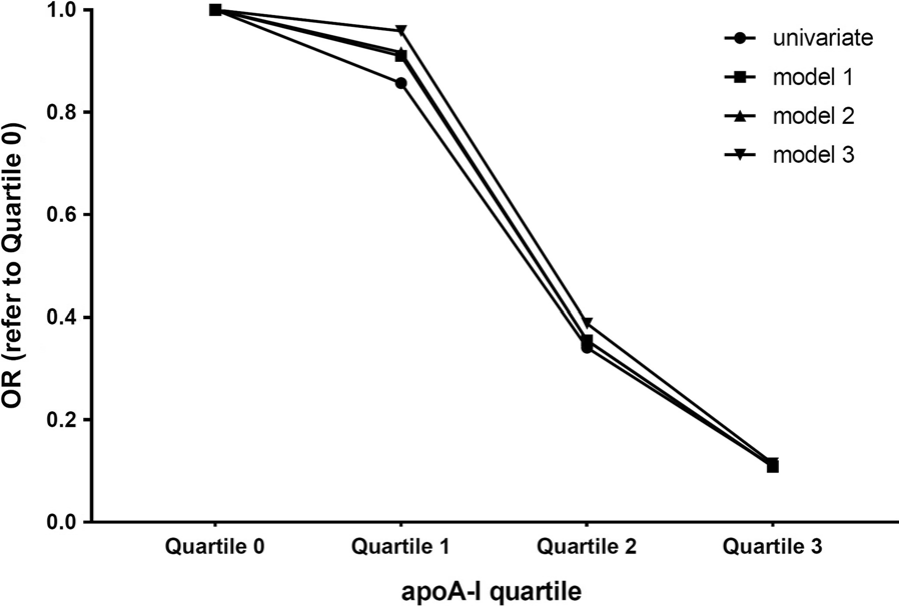
Association of each apoA-I quartile with late ISR incidence after PS matching, adjusted by three models

Quartile analysis illustrated a significant negative linear correlation between the incidence of late ISR and apoA-I. With increasing quartile of apoA-I, the incidence of late ISR decreased by 14.3%, 65.9% and 89% respectively, compared with the lowest quartile. Moreover, the linear correlation persisted even after adjusting for different confounding factors in the three clinical models (Table 6, Fig. 7).

**Table 6:** Quartile analysis of apoA-I associated with the incidence of late ISR after PS matching

apoA-I	0–25%	25–50%	50–75%	75–100%	*P* for trend
Quartile	0	1	2	3	
Mean	0.96 ± 0.13	1.21 ± 0.05	1.35 ± 0.06	1.64 ± 0.15	
Num	22	23	21	22	
Non-ISR	6	7	11	17	
ISR	16	16	10	5	
OR	1.00 (ref)	0.857	0.341	0.110	0.005*
Model 1	1.00 (ref)	0.910	0.355	0.109	0.010*
Model 2	1.00 (ref)	0.917	0.355	0.108	0.011*
Model 3	1.00 (ref)	0.959	0.388	0.115	0.015*

Model 1: adjusted for age, sex

Model 2: adjusted for age, sex, TC, TG, LDL-C

Model 3: adjusted for age, sex, TC, TG, LDL-C, hypertension, diabetes, smoking

### Association of apoA-I with all-patients ISR

Finally, all 604 patients enrolled in the study were divided into ISR group (n = 105) and non ISR group (n = 499). There were significant differences between the two groups with respect to baseline clinical characteristics and laboratory parameters. Therefore, propensity score matching (PSM) was again performed in a 1:1 ratio, using sex, age, stenting time, LDL-C, hypertension, diabetes, and smoking as matching variables, and 0.05 as the calipers. As a result, a propensity score-matched case group (ISR-PSM, n = 100) and control group (NISR-PSM, n = 100) were obtained to further evaluate the association of apoA-I with all-patients ISR.

After PSM, ISR patients had significantly longer stenting time (33.5 vs. 12, *P* = 0.008), while there were no significant differences with respect to the other clinical or laboratory features (Table 7).

**Table 7:** Patient characteristics and clinical profiles of all-patients PSM

Variants	ISR-PSM	NISR-PSM	*P* value
	N = 100	N = 100	
Male	77 (77%)	86 (86%)	0.101
Age	67.05 ± 9.96	66.92 ± 10.32	0.634
Hypertension	70 (70%)	71 (71%)	0.877
Diabetes	47 (47%)	58 (58%)	0.120
Old MI	25 (25%)	27 (27%)	0.747
Smoking	26 (26%)	36 (36%)	0.120
Total cholesterol	3.72 ± 1.33	3.73 ± 1.41	0.848
Triglyceride	1.73 ± 1.05	1.71 ± 1.09	0.955
LDL-C	2.37 ± 1.29	2.41 ± 1.21	0.823
HDL-C	1.03 ± 0.26	1.03 ± 0.28	0.518
apoA-I	1.15 ± 0.2	1.18 ± 0.22	0.314
apoB	0.85 ± 0.37	0.83 ± 0.34	0.707
apoE	38.07 ± 16.64	35.95 ± 18.05	0.543
Lp(a)	44.04 ± 64.48	39.57 ± 52.79	0.875
HbA1c	7.06 ± 1.63	7.36 ± 1.89	0.216
Hs-CRP	5.34 ± 11.02	5.77 ± 12.58	0.994
Creatine	81.92 ± 26.36	82.24 ± 22.09	0.931
eGFR	84.64 ± 21.53	85.21 ± 20.83	0.860
LVEF	58.13 ± 9.74	59.37 ± 6.88	0.562
Stenting time	33.5 (21–84)	12 (12–48)	0.008
Stent number	1.66 ± 0.8	1.72 ± 0.98	0.733
Stent diameter	2.72 ± 0.41	2.82 ± 0.38	0.139
Stent length	28.17 ± 7.04	26.92 ± 7.64	0.297

On multivariate logistic regression analysis of all-patients PSM cohort, apoA-I (OR 0.024, 95% CI 0.000–0.564), male sex (OR 2.674, 95% CI 1.041–6.870), and stenting time (OR 1.011, 95% CI 1.003–1.019) were independent risk factors for all-patients ISR (Table 8).

**Table 8:** Results of logistic regression analysis of all-patients PSM

Variants	Univariate			Multivariate		
	*P* value	OR	95% CI	*P* value	OR	95% CI
Male	0.104	1.835	(0.882–3.816)	0.041*	2.674	(1.041–6.870)
Age	0.927	1.001	(0.974–1.029)	0.221	0.976	(0.940–1.015)
Hypertension	0.732	0.889	(0.453–1.742)	0.597	1.220	(0.584–2.549)
Diabetes	0.546	0.833	(0.461–1.507)	0.589	0.793	(0.342–1.838)
Old MI	0.728	0.886	(0.447–1.754)	/		
Smoking	0.260	0.694	(0.368–1.310)	0.174	0.580	(0.264–1.271)
Total cholesterol	0.954	0.994	(0.811–1.218)	0.404	2.605	(0.275–24.681)
Triglyceride	0.873	1.022	(0.788–1.325)	0.718	1.143	(0.555–2.355)
LDL-C	0.833	0.976	(0.781–1.220)	0.373	0.365	(0.040–3.356)
HDL-C	0.815	0.886	(0.320–2.453)	0.311	5.446	(0.206–144.129)
apoA-I	0.376	0.520	(0.122–2.211)	0.024*	0.013	(0.000–0.564)
apoB	0.604	1.253	(0.534–2.938)	/		
apoE	0.427	1.007	(0.990–1.025)	/		
Lp(a)	0.616	1.001	(0.996–1.006)	/		
HbA1c	0.139	0.879	(0.741–1.043)	0.678	1.053	(0.826–1.341)
hs-CRP	0.803	0.997	(0.972–1.022)	/		
Creatine	0.930	0.999	(0.987–1.012)	/		
eGFR	0.859	0.999	(0.985–1.013)	/		
LVEF	0.559	0.979	(0.912–1.051)	/		
Stenting time	0.008*	1.009	(1.002–1.016)	0.010*	1.011	(1.003–1.019)

## Discussion

In this retrospective case–control study, apoA-I was found to be an independent risk factor for ISR in patients undergoing DES-based PCI. This finding is consistent with previous studies that demonstrated anti-restenosis effects of apoA-I. However, most previous studies on the anti-restenosis effects of apoA-I were conducted in cell and animal models. Aart et al. found that anti-apoA-I antibody coating metal stents inhibited ISR compared with bare metal stents in their in vitro experiments and in vivo iliac artery injury model of New Zealand rabbits [22]. In the study by Laura et al., apoA-I coated bare metal scaffolds were found to inhibit in-stent thrombosis, platelet activation, and SMC proliferation in vitro, and to improve the biocompatibility of the stents in a mice model [23]. Ben found that the increase of apoA-I and HDL-C, due to inhibition of cholesterol ester transfer protein (CETP), inhibited the proliferation of SMC and neointimal hyperplasia in New Zealand rabbit iliac artery injury model [15]. In the study by Liu et al., direct intragastric injection of apoA-I mimetic peptide inhibited the proliferation of SMC and neointimal hyperplasia in rat carotid artery injury model [24].

However, there is a paucity of clinical data regarding the association between apoA-I and ISR. Few clinical studies have suggested a significant difference of apoA-I between ISR and non-ISR patients, which is probably due to individual differences or discrepancy of treatment compliance. To the best of our knowledge, the present study is the first to indicate that apoA-I is an independent risk factor for ISR, as we eliminated the influence of most confounding factors by distinguishing early ISR from late ISR, by excluding complicated lesions (CTO, bifurcation lesion, long lesion, grafts lesion), by performing stratification according to lipid-lowering therapy, and by applying propensity score matching.

In this study, the overall incidence of ISR was 17.4% (early ISR 8.8% and late ISR 21.8%), which is consistent with previous reports (3–20%) [25, 26], suggesting that our study is clinically representative even in the era of new-generation DES.

In the early ISR study, we found no significant difference in LDL-C level between ISR and non-ISR patients, suggesting that lipid infiltration may not be the main pathophysiological mechanism of early ISR; this is in line with the current view that the main mechanisms of early ISR involve endothelial deprivation, neointimal hyperplasia, and SMC proliferation [4–7]. We observed a negative correlation of the incidence of early ISR with serum level of apoA-I, but not HDL-C, suggesting that apoA-I may inhibit neo-intimal hyperplasia or SMC proliferation, thereby inhibiting the occurrence and development of early ISR in an HDL-C independent manner.

In the late ISR study, although intensive lipid-lowering therapy (LDL-C ≤ 1.8 mmol/L) was associated with reduced incidence of ISR, 15.7% of the patients still progressed to late ISR, indicating that further reducing the incidence of late ISR is still clinically relevant. The current European Society of Cardiology (ESC) dyslipidemias management guidelines recommend reduction in LDL-C level to 1.4 mmol/L in high-risk patients with atherosclerotic cardiovascular disease (ASCVD) [27]. However, the mechanism of late ISR mainly involves neo-atherosclerosis, which is not exactly the same as that of natural atherosclerosis; therefore, large-scale clinical studies are required to determine whether further decrease in LDL-C reduces late ISR. In our study, apo A-I affected the incidence of late ISR similar to early ISR, suggesting potential anti-neo-atherosclerosis/anti-restenosis effects of apoA-I. Therefore, based on intensive LDL-C lowering, further increasing the apoA-I level may be a new therapeutic target to inhibit the occurrence and development of late ISR.

In the all-patients ISR study, apoA-I was found to be an independent risk factor for ISR and had a protective effect, which was consistent with the results of early ISR and late ISR study. However, we found that the median stenting time in the ISR group was 33.5 months in contrast to 12 months in the non-ISR group; this indicated that the ISR group contained most patients with late ISR, while most of the patients in the non-ISR group were those who were routinely followed-up. Therefore, although appropriate matching was carried out according to propensity score, different pathogenic mechanisms between early and late ISR may affect the results as a potential confounding factor.

The mechanism of the effect of apoA-I on ISR is likely complex and multifactorial. As the main protein component of HDL-C, apoA-I is mainly involved in the process of reverse transport of cholesterol. It helps remove fat and cholesterol from macrophages in the arterial wall, thereby reducing the transformation of macrophages into foam cells and inhibiting the progression of atherosclerosis [28]. Moreover, apoA-I also ameliorates vascular endothelial function, inhibits smooth muscle proliferation and platelet function, and reduces inflammation and angiogenesis in plaque as well as prevents native and neo-atherosclerosis [13–19]. Laura et al. investigated the effect of apoA-I on ISR through in vivo experiments in mice [14]. They found that apoA-I improved the biocompatibility of implanted stents. Injection of apoA-I inhibited neointimal hyperplasia after stenting in mice, which was due to the phenotypic transformation of neointima and the promotion of endothelialization [14]. Zhang and Mao, respectively, showed that apoA-I, in conjunction with apoA-I binding protein (AIBP), can enhance cholesterol reverse transport, inhibit inflammation and angiogenesis, and decrease the development of neo-atherosclerosis [29–33].

However, some limitations of our study should be acknowledged: (1) This was a single center, cross-sectional, retrospective case–control study. Owing to the lack of follow-up data, we could not assess the impact of dynamic changes of apoA-I on ISR. (2) Most patients in the early ISR study had undergone regular angiographic follow-up, while most patients in the late ISR study were re-hospitalized due to angina pectoris. As a result, the incidence of late ISR in this study may be higher than that of natural restenosis; therefore, our results may have been affected by an element of selection bias. (3) Although our study excluded patients with early-onset ISR within 6 months mainly due to operative factors (such as stenting with geography-lack or mal-apposition, or excessive elastic rebound caused by over-pressure post-dilation), the confounding bias due to other operative factors cannot be ruled out. (4) With no prior IVUS or OCT processed, the diagnosis of ISR may have been confused with late in-stent thrombosis, leading to some information bias. (5) Not all potential confounding factors were accounted for in the analysis due to the lack of data.

## Conclusions

In this study, serum level of apoA-I was an independent risk factor for ISR in patients undergoing DES-based PCI. Our results demonstrate that apoA-I may have an independent protective effect against early and late ISR regardless of HDL-C level. Further multi-center cohort studies and randomized controlled trials are required to provide more definitive evidence of apoA-I as a promising therapeutic target for further reducing the incidence of ISR after DES-based PCI.

## Data Availability

The datasets generated and analyzed during the present study are available from the corresponding author on reasonable request.

## Abbreviations

DES: Drug-eluting stents
ISR: In-stent restenosis
PCI: Percutaneous coronary intervention
MACE: Major adverse cardiac events
IVUS: Intravascular ultrasound
OCT: Optical coherence tomography
SMC: Smooth muscle cell
CTO: Chronic total occlusion
QCA: Quantitative coronary angiography
TC: Total cholesterol
TG: Triglyceride
LDL-C: Low density lipoprotein cholesterol
HDL-C: High density lipoprotein cholesterol
apoA-I: Apolipoprotein A-I
apoB: Apolipoprotein B
apoE: Apolipoprotein E
Lp(a): Lipoprotein(a)
HbA1c: Glycosylated hemoglobin
hs-CRP: High-sensitivity C-reactive protein
eGFR: Estimated glomerular filtration rate
LVEF: Left ventricular ejection fraction
ROC: Receiver operating characteristic
AUC: Area under the curve
NRI: Net reclassification index
IDI: Integrated discrimination improvement
PSM: Propensity score matching
CETP: Cholesterol ester transfer protein
ESC: European Society of Cardiology
ASCVD: Atherosclerotic cardiovascular disease
AIBP: ApoA-I binding protein
LAD: Left anterior descending coronary artery
LCX: Left circumflex coronary artery
RCA: Right coronary artery
BVS: Biodegradable vascular scaffold
SVG: Saphenous vein graft
ACEI: Angiotensin converting enzyme inhibitor
ARB: Angiotensin receptor blocker
MI: Myocardial infarction
